# Response to imatinib rechallenge in a patient with a recurrent gastrointestinal stromal tumor after adjuvant therapy: a case report

**DOI:** 10.1186/1752-1947-5-504

**Published:** 2011-10-05

**Authors:** Yoon-Koo Kang

**Affiliations:** 1 Department of Oncology, University of Ulsan College of Medicine, Asan Medical Center, Seoul, Korea

## Abstract

**Introduction:**

Adjuvant imatinib improves recurrence-free survival of patients following resection of primary KIT-positive gastrointestinal stromal tumors. However, it is unknown whether patients who previously received adjuvant imatinib therapy will respond to imatinib rechallenge as treatment for recurrent disease. Here we present the first report documenting the benefits of imatinib rechallenge in a patient previously exposed to imatinib during adjuvant treatment.

**Case presentation:**

A 51-year-old Asian woman with a wedge-resected primary gastric gastrointestinal stromal tumor at high risk of relapse underwent two years of adjuvant treatment with imatinib. Only 10 months after the completion of adjuvant imatinib treatment, a computed tomography scan revealed gastrointestinal stromal tumor recurrence in this patient, with multiple peritoneal nodules in the upper abdomen being detected. Our patient was rechallenged with imatinib 400 mg/day and had a partial response after one month of treatment. Imatinib rechallenge was well tolerated by our patient; the only adverse events she experienced were grade 1 edema, anemia and fatigue. Our patient maintained a partial response two years and six months after the imatinib rechallenge. However, computed tomography scans three months later showed that our patient had disease progression.

**Conclusions:**

This case report demonstrates that a patient with a gastrointestinal stromal tumor who had previously received adjuvant imatinib therapy responded to imatinib rechallenge as treatment for her recurrent disease. These results indicate that imatinib sensitivity can be maintained in a patient with previous exposure to adjuvant imatinib therapy.

## Introduction

Gastrointestinal stromal tumors (GIST) are the most common mesenchymal tumors of the gastrointestinal tract. These tumors are characterized by activating mutations of either receptor tyrosine kinase KIT or, less commonly, platelet-derived growth factor receptor α (PDGFRα) [1]. Imatinib mesylate, an oral selective inhibitor of KIT and PDGFRα, is approved for the treatment of adult patients with unresectable and/or metastatic KIT-positive (KIT+) GIST. It is also indicated for the adjuvant treatment of adult patients following resection of GIST [2]. Current guidelines [3,4] recommend adjuvant treatment with imatinib for at least one year and the use of risk assessment systems based on the main variables of mitotic rate, tumor size, tumor site and tumor rupture to guide patient selection for adjuvant imatinib therapy. This recommendation is based on results from the pivotal American College of Surgeons Oncology Group (ACOSOG) Z9001 trial. This trial demonstrated a significant one-year recurrence-free survival (RFS) benefit for adjuvant imatinib versus placebo after the treatments were given for one year in patients at various levels of risk for recurrence [5]. However, optimal duration of adjuvant imatinib has not yet been determined, and a longer course of therapy may be needed for patients at higher risk of recurrence.

A recent Korean, single-arm, Phase II study evaluated the efficacy of two years of adjuvant imatinib treatment in GIST patients with *KIT *exon 11 mutations who are at high risk of relapse following surgical resection of the primary GIST [6]. The study showed a two-year RFS rate of 93.3%, which compared favorably with an RFS of 73% at two years after one year of adjuvant imatinib therapy in a similar patient population, reported in another single-arm, Phase II study (ACOSOG Z9000) [7]. These results suggest that a longer duration of adjuvant imatinib treatment may improve the RFS of patients with KIT+ GIST after surgery. However, it is unclear whether this improvement in RFS will ultimately extend overall survival (OS) in these patients. One of the critical factors that will affect OS improvement is the effect of adjuvant therapy on the efficacy of imatinib rechallenge for treating recurrent disease. Although the results of another trial (BFR14) showed that most patients with progressive metastatic GIST were able to respond to imatinib rechallenge after treatment interruption [8], it remains to be determined whether imatinib sensitivity could also be maintained after completion of adjuvant treatment. Here, we report on a patient who responded to rechallenge with imatinib after experiencing recurrence subsequent to completion of two years of adjuvant imatinib therapy.

## Case presentation

A 51-year-old Asian woman diagnosed with a primary gastric GIST underwent wedge resection, achieving complete removal of the entire tumor with microscopic examination of the margins showing no tumor cells (R0 margins). She was considered at high risk for recurrence based on the tumor size (8 cm × 7 cm × 3 cm) and high mitotic rate (>50 mitoses per 50 high powered fields (HPF)) of the excised GIST [9,10]. In addition, mutation analysis showed that the tumor had *KIT *exon 11 deletions, a genotype shown to be associated with adverse outcomes after surgery [10]. As such, our patient was enrolled in the aforementioned Korean Phase II trial of adjuvant imatinib for patients with localized *KIT *exon 11-mutant GIST at high risk of relapse [6]. Our patient was started on imatinib adjuvant treatment 400 mg/day but developed a skin rash after three months; the rash was successfully managed with a temporary (three-week) dose interruption. Our patient was subsequently restarted on imatinib 300 mg/day and resumed taking standard-dose imatinib 400 mg/day after three months. She was maintained, generally tolerated this dose and completed the two-year adjuvant therapy regimen.

**Figure 1:**
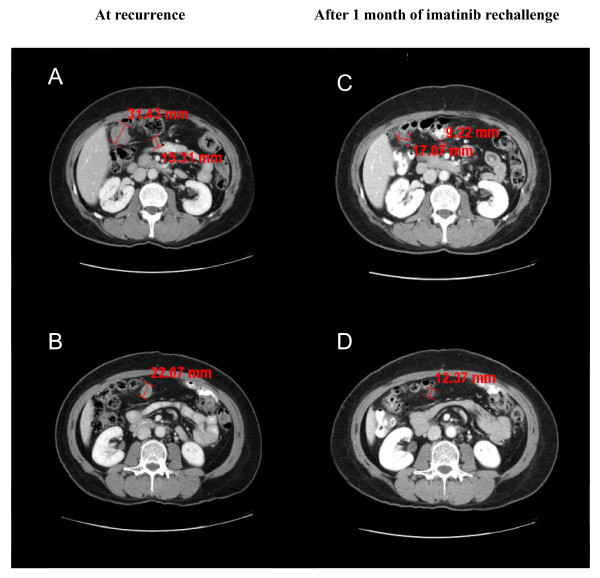
**CT scans of our patient before and after imatinib rechallenge**. **(A,B) **Recurrent tumors (nodules) can be seen in the peritoneum of our patient, who had received prior adjuvant imatinib treatment. **(C,D) **After one month of rechallenge with imatinib, our patient demonstrated a partial response to imatinib treatment, with the nodule sizes decreasing by 43%.

Ten months after stopping adjuvant imatinib treatment, recurrence was detected in this patient, as computed tomography (CT) scans revealed three gross peritoneal nodules in her upper abdomen (Figure 1A and 1B). Our patient was rechallenged with imatinib 400 mg/day as first-line treatment for her recurrent or metastatic disease. After one month of treatment, a partial response (PR) by Response Evaluation Criteria In Solid Tumors was observed; the three peritoneal nodules had decreased in size from 31.4 mm, 15.3 mm and 22.1 mm (sum, 69 mm; Figure 1A and 1B) to 17.1 mm, 9.2 mm and 12.4 mm, respectively (sum, 39 mm; Figure 1C and 1D), representing a 43% decrease in size. Imatinib rechallenge was well tolerated, as the only adverse events experienced by our patient were grade 1 edema, anemia and fatigue. Our patient maintained a stable PR for over two and a half years after being rechallenged with imatinib treatment, as evidenced by repeated CT scans. However, progression was observed three months later; CT scans revealed that the sum of two peritoneal nodules had increased in size by 50% since the last tumor assessment. Disease progression was confronted through dose escalation to imatinib 800 mg/day. Our patient's response to dose escalation will be monitored closely during future follow-up visits.

## Discussion

The results of this case report demonstrate that a GIST patient who has undergone adjuvant imatinib therapy responded when rechallenged with imatinib as a treatment for her recurrent disease. These results suggest that sensitivity to imatinib was not compromised by prior exposure to adjuvant imatinib.

The Korean adjuvant Phase II study referred to in this case report enrolled patients with resected primary GIST possessing *KIT *exon 11 mutations at high risk of recurrence (mitotic rate ≥5 mitoses/50 HPF and tumor size ≥5 cm, or mitotic rate ≥10 mitoses/50 HPF, or tumor size ≥10 cm) [9,10]. Our previous retrospective study showed that the presence of *KIT *mutations, along with a high mitotic rate and larger tumor size, was an independent risk factor for poor prognosis in patients with localized GIST [10]. Because GIST harboring *KIT *exon 11 mutations appeared to be more sensitive to imatinib treatment than GIST of other genotypes in the metastatic setting [11] and, shown more recently, in the adjuvant setting [12], patients with *KIT *exon 11 mutations at high risk of recurrence would be most likely to benefit from adjuvant treatment with imatinib. The fact that some patients, including our patient described in this case report, developed disease recurrence after stopping adjuvant therapy suggests that two years of adjuvant imatinib treatment may not be sufficient for eradicating residual GIST tumor cells and preventing relapse in these high-risk patients. Our patient's residual tumor cells remained sensitive to imatinib therapy, as evidenced by her good response to subsequent imatinib rechallenge. However, it appears that the sensitive residual tumor cells need to be continuously suppressed with adjuvant imatinib therapy to prevent or further delay disease recurrence. The question of how long patients should be treated with adjuvant imatinib remains. The Phase III study conducted by the Scandinavian Sarcoma Group (SSG) and the Sarcoma Group of the Arbeitsgemeinschaft Internistische Onkologie (AIO; SSGXVIII/AIO study) recently demonstrated that three years of adjuvant imatinib treatment, compared with one year of imatinib treatment, significantly improves RFS and OS in GIST patients who have a high estimated risk of recurrence after surgery [13]. This suggests that patients at high risk of recurrence, such as our patient described in this case report, should receive adjuvant imatinib treatment for a minimum duration of three years. This is reflected in the updated National Comprehensive Cancer Network guidelines that, based on the results of the SSGXVIII/AIO trial, recommend considering adjuvant imatinib for at least 36 months for patients with high-risk GIST [14].

Our patient in this case report remained progression-free for two years and nine months, which is shorter than the median progression-free survival (PFS) of approximately four years achieved in a Korean Phase II study of patients with advanced GIST [15], but longer than the median PFS of 18 to 20 months reported in other studies of imatinib therapy for metastatic or recurrent GIST [16]. Although it is not feasible to compare the results of clinical studies conducted in different patient populations at different times, the 33 months of PFS we observed in this case report suggest that our patient maintained sensitivity to imatinib even though she was previously exposed to imatinib for two years in the adjuvant setting.

## Conclusion

Rechallenge with imatinib may provide substantial clinical benefit to patients with recurrent GIST after cessation of adjuvant imatinib therapy.

## Consent

Written informed consent was obtained from our patient for publication of this case report and any accompanying images. A copy of the written consent is available for review by the Editor-in-Chief of this journal.

## Competing interests

YKK is a consultant for Novartis, and has received research funding and honoraria from Novartis for lectures.
